# Euthyroid Sick Syndrome as a Predictor of Hospital Stay in Pediatric Diabetic Ketoacidosis

**DOI:** 10.3390/jcm15072501

**Published:** 2026-03-25

**Authors:** Youssef A. Alqahtani, Ayed A. Shati, Ayoub A. Alshaikh, Abdulrahman Hassan Nasser Alasmari, Fahad Aedh Alghamdi, Noura Abdulrahman Alamri, Sarah Ibraheem Summan, Oroub Mohammed Amir Atif, Ramy Mohamed Ghazy

**Affiliations:** 1 Department of Child Health, College of Medicine, King Khalid University (KKU), Abha 61421, Saudi Arabia; youssef98118911@gmail.com (Y.A.A.); shatiayed@gmail.com (A.A.S.); 2 Family and Community Medicine Department, College of Medicine, King Khalid University (KKU), Abha 61421, Saudi Arabia; alashaikh@kku.edu.sa; 3 Pediatric Emergency Department, Abha Maternity and Children Hospital, Abha 61421, Saudi Arabia; abhalasmri@moh.gov.sa (A.H.N.A.); falghamdi127@moh.gov.sa (F.A.A.); 4 Aseer Health Cluster, Ministry of Health, Abha 61421, Saudi Arabia; nalamri24@moh.gov.sa (N.A.A.); saraibraheem@hotmail.com (S.I.S.); mohoroub@gmail.com (O.M.A.A.); 5 Tropical Health Department, High Institute of Public Health, Alexandria University, Alexandria 21561, Egypt

**Keywords:** euthyroid sick syndrome, pediatric diabetic ketoacidosis, complications, hospital stay, thyroid function, Kaplan–Meier, cox regression

## Abstract

**Background:** Euthyroid sick syndrome (ESS) is commonly diagnosed in children during acute metabolic stress such as diabetic ketoacidosis (DKA). Nevertheless, the association of ESS with clinical outcomes has not been fully established. This study aimed to address the association between ESS and duration of hospital stay among pediatric patients presenting with DKA. **Methods:** This retrospective cohort study included 176 children admitted with confirmed DKA. Baseline clinical, biochemical, and outcome data, including complications and time to discharge, were collected. Kaplan–Meier survival analysis and Cox proportional hazards regression models were used to assess factors associated with duration of hospital stay. **Results:** Children were classified based on thyroid function tests at admission into ESS (*n* = 112, 63.6%) and non-ESS (*n* = 64, 36.4%). Children with ESS were younger [median age 10.0 (6.5–13.5) years vs. 14.0 (11.5–16.0) years; *p* < 0.001], had lower median weight [31.0 (20.5–44.5) Kg vs. 40.5 (34.5–49.5) Kg; *p* < 0.001], had lower median BMI [18.0 (16.5–20.0) kg/m^2^ vs. 19.0 (17.5–20.5) kg/m^2^; *p* = 0.007), and slightly lower mean pH at admission [7.1 ± 0.1 vs. 7.2 ± 0.1, *p* = 0.016]. Free T3 (2.4 (2.0–3.4) vs. 5.1 (4.2–5.5) pmol/L), Free T4 (12.0 (10.7–14.1) vs. 14.4 (14.0–16.2) pmol/L), and TSH 1.8 (1.1–2.9) vs. 2.7 (1.7– 3.2) mIU/L) were significantly lower in ESS patients (*p* < 0.001 for all). Impaired consciousness occurred exclusively in the ESS group (8.9% vs. 0%, *p* = 0.034). Median hospital stay was longer among ESS patients, with over a quarter hospitalized for ≥5 days (26.8% vs. 0%; *p* < 0.001). Kaplan–Meier analysis showed significantly prolonged hospitalization for ESS patients (log-rank *p* < 0.0001). Patients with ESS [hazard ratio (HR) = 0.31; 95% CI, 0.21–0.45; *p* < 0.001], pediatric intensive care unit admission [HR = 0.49; 95% CI, 0.29–0.83; *p* = 0.008], moderate DKA [HR = 0.51; 95% CI, 0.30–0.87; *p* = 0.014], and severe DKA [HR = 0.28; 95% CI, 0.14–0.57; *p* < 0.001] were associated with prolonged hospital stay. **Conclusions:** ESS is significantly associated with prolonged hospital stays in children with DKA. Early identification of ESS may help guide monitoring strategies and discharge planning.

## 1. Introduction

Worldwide, type 1 diabetes mellitus (T1DM) is a common endocrine disorder that affects children and adolescents. Although symptoms typically appear during childhood or adolescence, disease onset can also occur later in adulthood [1]. T1DM is associated with multiple acute and chronic complications [2]. Subjects with T1DM have roughly a 12-year life expectancy shorter than the general population [3]. This autoimmune disease is characterized by the destruction of pancreatic β-cells, leading to insulin deficiency and sustained hyperglycemia [1]. The principal susceptibility locus is located within the HLA region on chromosome 6, with notable associations to the alleles DR3, DR4, DQA1×0501, DQB1×0201, DQA1×0301, and DQB1×0302. Variants within the HLA complex account for approximately 40–50% of the total genetic risk for developing T1DM [4].

In recent decades, a pronounced change in both the prevalence and incidence of T1DM has been documented in Saudi Arabia, particularly among children and adolescents [5,6,7]. According to estimates from the International Diabetes Federation (IDF) in 2024, Saudi Arabia is one of the countries with the highest diabetes burdens in the region. Approximately 222,942 individuals are living with T1DM, including 35,000 children and adolescents. Globally, Saudi Arabia ranks among countries with the highest incidence of pediatric T1DM, reaching approximately 33.5 cases per 100,000 children per year [8]. These high figures underscore a growing public health challenge and highlight the urgent need for enhanced surveillance systems, earlier detection strategies, and comprehensive diabetes care across the country.

Euthyroid sick syndrome (ESS) refers to abnormal thyroid function test results in the absence of any intrinsic dysfunction of the hypothalamus, pituitary gland, or thyroid gland [9]. ESS is characterized by reduced levels of free triiodothyronine (FT3) and total triiodothyronine (TT3), an elevation in reverse T3 (rT3), variable changes in thyroid-stimulating hormone (TSH), and thyroxine (T4) levels that may stay normal or decline in more severe cases [10,11]. Interestingly, T4 levels below 4 μg/dL and 2 μg/dL are associated with around 50% and 80% mortality, respectively. Additional evidence suggests that a decrease in T3 levels and an increase in rT3 levels are independent predictors of patient survival [9,12].

Diabetic ketoacidosis (DKA) represents a state of severe metabolic stress; it is usually associated with alterations in thyroid hormone levels. These hormonal disturbances are most likely due to ESS rather than intrinsic thyroid dysfunction [11]. Even mild metabolic disturbances, such as slight acidosis combined with hyperglycemia, can progress to more severe derangements [10]. Several studies showed that about three-fifths of children with DKA can develop ESS. Moreover, an inverse association between acidosis and concentrations of free thyroid hormones has been reported [13,14]. ESS is proposed to develop in the setting of DKA through several mechanisms, including dysregulation of the hypothalamic–pituitary–thyroid axis, the effects of inflammatory cytokines, and oxidative stress [15].

Although ESS has been associated with poor outcomes in other critical health conditions, including myocardial infarction [16], severe acute respiratory syndrome coronavirus 2 (SARS-CoV2) disease [17], and multisystem inflammatory syndrome in children (MIS-C) [18], it is unclear whether it represents an adaptive physiological response or a predictor of complications in children with DKA. Among adults with DKA, ESS was found to be significantly associated with long duration of hospital stay and higher hospitalization cost compared with those without ESS [19]. However, the literature assessing the relationship between ESS and the duration of hospital stay in the pediatric population remains limited. Studying the association between ESS at presentation and subsequent clinical outcomes, including the length of hospitalization, is essential for effective risk stratification and optimizing patient management. Therefore, this study aimed to evaluate the association between ESS, and the duration of hospital stay in children with DKA.

## 2. Materials and Methods

### 2.1. Study Design

This retrospective cohort study was conducted from 1 January 2023 to 30 June 2025 through chart review. The study was conducted at Pediatric Endocrinology and Emergency Departments at two tertiary care centers—Abha Maternity and Children Hospital and King Khalid University Medical City. The study design and reporting follow the Strengthening Reporting of Observational Studies in Epidemiology (STROBE) guidelines [20].

### 2.2. Study Participants

The study included pediatric patients with T1DM presenting with DKA. Patients were stratified into two groups according to their thyroid function tests obtained at admission: those exhibiting ESS and those with normal thyroid function. Clinical characteristics, DKA severity, and hospital stay duration were compared between groups.

Inclusion Criteria:

- Age ≤ 18 years.
- Newly diagnosed or previously known T1DM, per ISPAD criteria (fasting plasma glucose ≥ 126 mg/dL, random glucose ≥ 200 mg/dL plus classic symptoms, i.e., polyuria, weight loss, or HbA1c ≥ 6.5% in the presence of autoantibodies, including glutamic acid decarboxylase, tyrosine phosphatase-like insulinoma antigen 2 autoantibodies, insulin autoantibodies, or zinc transporter 8 autoantibodies) [21].
- DKA was defined according to the following biochemical criteria: hyperglycemia (blood glucose > 200 mg/dL), metabolic acidosis (venous pH < 7.3 or serum bicarbonate < 15 mmol/L), and presence of ketosis, either serum β-hydroxybutyrate ≥ 3.0 mmol/L or moderate to large urine ketones [22].
- Availability of thyroid function tests at DKA presentation (TSH, free T3, free T4) drawn before insulin therapy or fluid resuscitation.

Exclusion criteria:

- Chronic systemic illnesses (e.g., chronic kidney disease, malignancy, liver disease).
- History of thyroid disease or thyroid hormone therapy.
- Medications affecting thyroid function (e.g., glucocorticoids, dopamine, phenytoin) in the last month.
- Non-diabetic ketoacidosis.
- Type 2 diabetes mellitus.
- Incomplete medical records.

### 2.3. Study Variables

#### 2.3.1. Dependent Variables (Outcomes)

The duration of hospitalization was measured in days from admission to discharge. Discharge was carried out according to a standardized institutional protocol. Patients were discharged after resolution of metabolic acidosis, defined as a venous pH ≥ 7.3 and a serum bicarbonate level ≥ 15 mmol/L, with successful transition from intravenous insulin infusion to a subcutaneous insulin regimen. In addition, clinical stability had to be completed, with the ability to tolerate oral intake for at least 24 h.

#### 2.3.2. Independent Variables

Demographics: age, sex, weight, height, and BMI. BMI was calculated as weight in kilograms divided by height in meters squared (kg/m^2^) and interpreted using age- and sex-specific WHO/CDC growth-chart percentiles, as BMI varies with normal growth and development in children. Based on these percentiles, participants were classified as underweight (<5th percentile), normal weight (5th–<85th percentile), overweight (85th–<95th percentile), or obese (≥95th percentile).

Clinical characteristics: consciousness (normal, or impaired), dehydration grade (based on clinical assessment): mild [3–5%], moderate [6–9%], severe [≥10%]), infection (clinical and/or laboratory-confirmed), pediatric intensive care unit (PICU) admission (yes/no), and electrolyte disturbances (yes/no).

Biochemical variables: blood glucose at diagnosis, glycated hemoglobin (HbA1c), venous blood gas (pH, bicarbonate), serum electrolytes (sodium, potassium, chloride), serum creatinine, and thyroid function tests (TSH, free T4, free T3).

### 2.4. Operational Definitions

Participants were categorized according to age-adjusted thyroid hormone level into ESS and non-ESS. Normal thyroid function was defined by values falling within the following ranges: for children < 1 year, TSH 0.8–8.2 mIU/L, FT4 11–23 pmol/L, and FT3 5.5–10.0 pmol/L; for those aged 1–6 years, TSH 0.7–6.0 mIU/L, FT4 14–26 pmol/L, and FT3 5.7–13.1 pmol/L; for 7–12 years, TSH 0.6–5.0 mIU/L, FT4 13–23 pmol/L, and FT3 4.5–10.0 pmol/L; and for adolescents ≥ 13 years, TSH 0.4–4.2 mIU/L, FT4 12–21 pmol/L, and FT3 3.5–9.4 pmol/L. ESS was diagnosed when FT3 was below the age-specific reference range, with TSH typically remaining within the normal limits for age. FT4 levels were either normal or slightly reduced [11,23].

DKA severity was classified at presentation based on biochemical parameters and clinical features, before starting treatment. Mild DKA was defined as venous pH ≥ 7.20 and serum bicarbonate ≥ 15 mmol/L. Moderate DKA was defined as venous pH 7.10–7.19 and serum bicarbonate 8–14 mmol/L. Severe DKA was defined as venous pH < 7.10 and serum bicarbonate < 8 mmol/L [24].

Level of consciousness was assessed using the Glasgow Coma Scale (GCS), which assesses three key aspects of neurological function: eye opening, verbal response, and best motor response, with a total score ranging from 3 to 15. Patients were categorized as either normal (scored 15) or abnormal (less than 15).

### 2.5. Data Collection

Data were extracted from medical records, including demographics, clinical presentation, DKA severity, thyroid function, complications, and hospitalization outcomes. Thyroid function was assessed at DKA presentation using age-specific reference ranges.

### 2.6. Quality Control and Data Management

To ensure data accuracy, double data entry was performed for 20% of randomly selected records, achieving an agreement rate above 90%. Any discrepancies were resolved by consensus or adjudicated by a senior author. All data extractors received formal training on the proper use of the data collection form and operational definitions. Missing values were recorded as “NA,” and variables with more than 10% missing values were removed. No imputation was made due to the low proportion of missing data for included variables.

### 2.7. Ethical Considerations

The study was conducted in accordance with the Declaration of Helsinki and complied with national regulations on human research. This study was approved by the King Khalid University Research Ethics Committee [HAPO-06-B-001; KKU-171-2025-31]. Due to the retrospective nature of the study, informed consent was waived. Despite being extracted from patient records, all data were anonymized and stored securely to maintain confidentiality. Only the principal investigator and the senior author had access to the data.

### 2.8. Statistical Analysis

Data normality was assessed using the Shapiro–Wilk test. Continuous variables were summarized as mean ± standard deviation (SD) when data were normally distributed, while median and interquartile range [IQR] were used when data normality was violated. Comparisons between patients with ESS and those without ESS were performed using the independent samples *t*-test for normally distributed continuous variables and the Mann–Whitney U test for non-normally distributed ones. Categorical variables were compared using the Chi-square test, while if test assumptions were violated, Fisher’s exact test was used instead. To assess the correlation between duration of hospital stay and clinical or biochemical variables (pH, HCO_3_, FT3, FT4, TSH, blood glucose, and HbA1c), Spearman correlation coefficients (r) were calculated for non-normally distributed data, while Pearson correlation coefficients were used for normally distributed variables. The strength of correlations was interpreted based on the absolute value of the correlation coefficient (r), <0.20 was considered negligible, 0.20–0.39 weak, 0.40–0.59 moderate, 0.60–0.79 strong, and ≥0.80 very strong. Survival analysis was conducted to evaluate the duration of hospitalization. Hospital discharge was defined as an event of interest, and patients remaining hospitalized were considered censored observations. Kaplan–Meier curves were used to evaluate the duration of hospitalization, with differences between the ESS and non-ESS groups assessed using the log-rank test. Univariate Cox proportional hazards regression was first performed to evaluate the crude association of ESS, age, PICU admission, HbA1c, and DKA severity with duration of hospital stay. Variables significant in univariate analysis or deemed clinically important were then included in multivariate Cox proportional hazards, with hazard ratios (HRs) less than 1, indicating a slower rate of discharge, corresponding to a longer hospital stay. The interaction between ESS and DKA severity was tested by including interaction terms in the Cox model. A likelihood ratio test compared models with and without interaction terms. The proportional hazards assumption was assessed using Schoenfeld residuals. For variables that violated the proportional hazards assumption, a sensitivity analysis was performed using a stratified Cox model, which allows for different baseline hazard functions across strata of the violating variable while providing adjusted hazard ratio estimates for the remaining covariates. Statistical significance was two-tailed with a *p*-value < 0.05. All analyses were performed using R version 4.3.0, leveraging key packages including dplyr for data manipulation, ggplot2 for visualization, and survival for time-to-event modeling.

### 2.9. Artificial Intelligence Use

During the preparation of this manuscript, the authors utilized ChatGPT-4.0 and DeepSeek solely for grammar correction, spelling verification, and minor stylistic improvements to enhance readability. No generative AI tools were used for data collection, statistical analysis, study design, or the generation of scientific conclusions. The authors reviewed all edits made by these tools and assumed full responsibility for the final content.

## 3. Results

The study included 176 children with DKA, of whom 112 (63.6%) had ESS and 64 (36.4%) had normal thyroid function. The participants’ median age was 12.0 years (IQR 9.0–15.0). ESS children were younger than non-ESS children [(10.0 (6.5–13.5) years vs. 14.0 (11.5–16.0) years; *p* < 0.001]. ESS patients also had lower median body weight [31.0 (20.5–44.5) kg vs. 40.5 (34.5–49.5) kg, *p* < 0.001] and median BMI [18.0 (16.5–20.0) kg/m^2^ vs. 19.0 (17.5–20.5) kg/m^2^, *p* = 0.007]. At admission, the ESS group presented with a marginally lower mean pH (7.1 ± 0.1 vs. 7.2 ± 0.1, *p* = 0.016). Thyroid function testing in the ESS group demonstrated significantly reduced FT3 [2.4 (2.0–3.4) pmol/L vs. 5.1 (4.2–5.5), *p* < 0.001], FT4 [12.0 (10.7–14.1) pmol/L vs. 14.4 (14.0–16.2) pmol/L, *p* < 0.001), and TSH [1.8 (1.1–2.9) mIU/L vs. 2.7 (1.7–3.2) mIU/L, *p* < 0.001). There were no statistically significant differences between-group in sex distribution, blood glucose, or HbA1c (Table 1).

**Figure 1:**
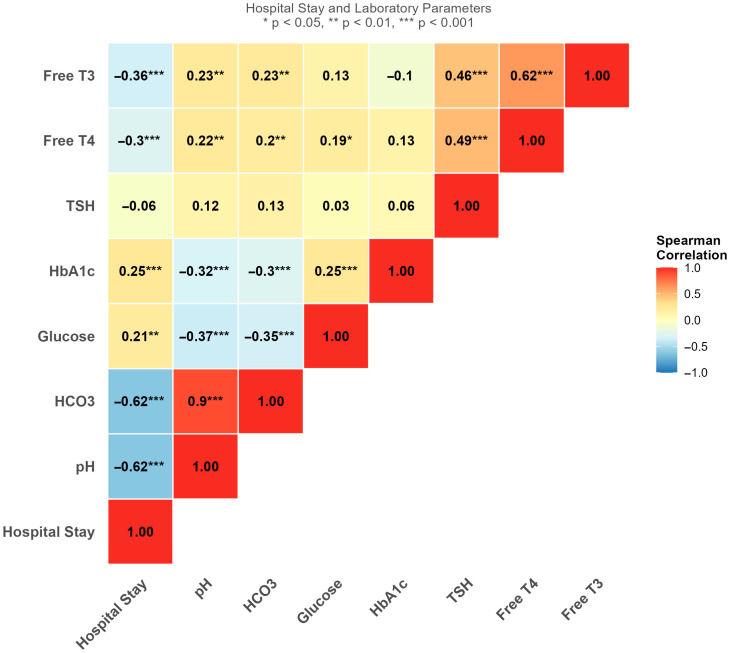
Correlation matrix of clinical, metabolic, and thyroid variables with duration of hospital stay in pediatric DKA.

Lower pH and HCO_3_ levels at admission were strongly associated with longer hospitalization (r = −0.62 for both, *p* < 0.001). Similarly, reduced FT3 and FT4 levels showed moderate negative correlations with hospital stay (r = −0.36 and r = −0.30, respectively; *p* < 0.001). In contrast, TSH demonstrated no significant correlation with hospital stay (r = −0.06). Blood glucose and HbA1c showed weak positive correlations with hospital stay (r = 0.21 and r = 0.25, respectively) (Figure 1).

All cases of impaired consciousness were exclusively in the ESS group, with 8.9% of ESS patients presenting with moderate or severe impairment of consciousness, compared to none in the non-ESS group (*p* = 0.034). Hospitalization was significantly longer for the ESS cohort (mean 4.1 ± 1.0 days vs. 3.3 ± 0.6 days, *p* < 0.001). None of the non-ESS group were hospitalized for more than 4 days, whereas 26.8% of ESS patients had extended stays of 5 days or more. Other clinical parameters, including dehydration grade, prevalence of electrolyte abnormalities, presence of concurrent infection, and PICU admission, showed no significant differences between groups. Reassuringly, there were no deaths in either group during the study period (Table 2).

**Figure 2:**
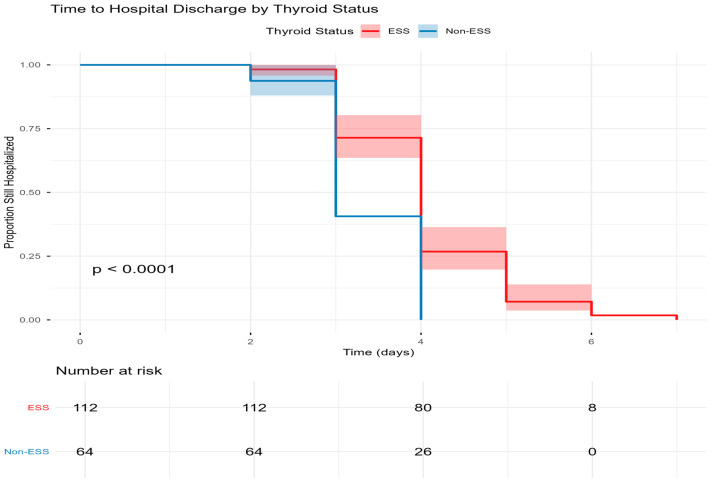
Kaplan–Meier curve of hospital stays among DKA patients with and without ESS.

The Kaplan–Meier survival analysis demonstrates that patients with ESS tend to have longer hospital stays compared to those with normal thyroid function. The log-rank test indicates a statistically significant difference between the two groups (*p* < 0.0001). On day 4, roughly 70.0% of ESS patients remain hospitalized compared to 40.0% of patients without ESS (Figure 2).

**Figure 3:**
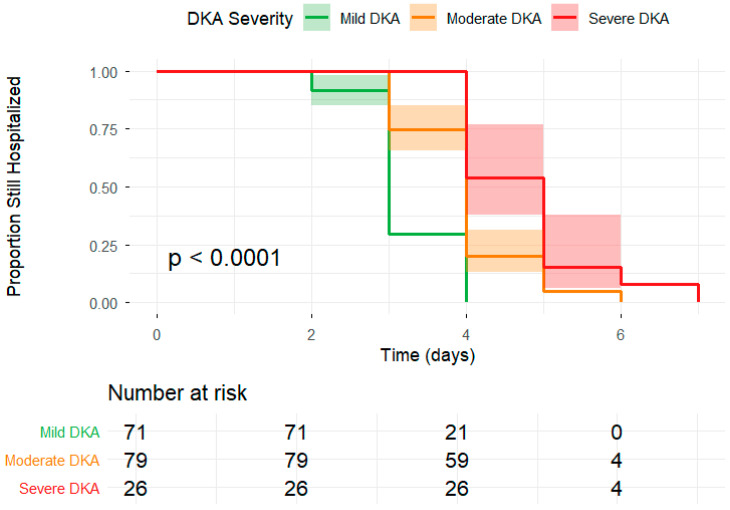
Thyroid status and severity of diabetic ketoacidosis significantly influence the duration of hospitalization in pediatric patients.

Patients with ESS, particularly those with moderate or severe DKA, experience significantly prolonged hospitalizations compared to those with mild DKA. The curves show that patients who had mild DKA were discharged earliest, with around 30.0% remaining hospitalized by day 4. In contrast, patients with moderate or severe DKA exhibit markedly delayed discharge beyond day 4 (75.0% and 100.0%, respectively) (Figure 3).

**Figure 4:**
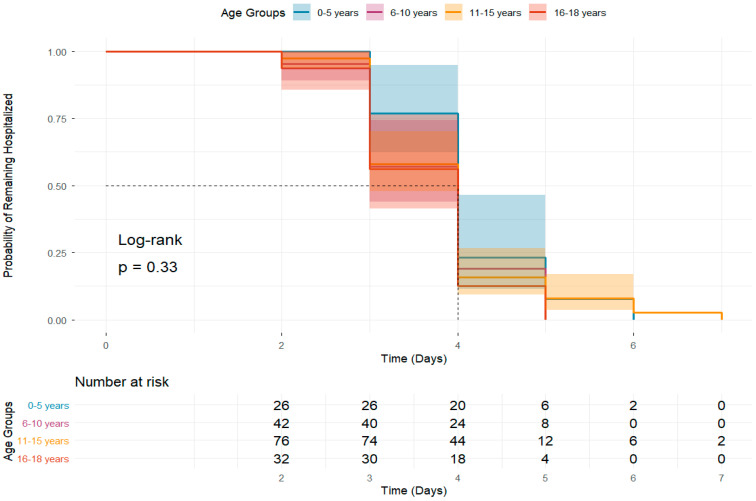
Duration of hospital stay among pediatric patients: a survival analysis by age group.

This Kaplan–Meier survival analysis examines the probability of remaining hospitalized over time across four pediatric age groups (0–5, 6–10, 11–15, and 16–18 years). The results indicate no statistically significant difference in hospital stay duration between the groups (log-rank *p* = 0.33) (Figure 4).

On univariate analysis, pediatric DKA patients with ESS had a significantly lower hazard of discharge compared with patients without ESS (HR = 0.39; 95% CI, 0.27–0.54; *p* < 0.001). PICU admission was also associated with a reduced discharge hazard (HR = 0.34; 95% CI, 0.25–0.48; *p* < 0.001). With respect to DKA severity, both moderate DKA (HR = 0.33; 95% CI, 0.23–0.46; *p* < 0.001) and severe DKA (HR = 0.16; 95% CI, 0.10–0.26; *p* < 0.001) were associated with prolonged hospitalization compared with mild DKA. In contrast, age (HR = 1.03; 95% CI, 0.99–1.07; *p* = 0.096) and HbA1c (HR = 0.92; 95% CI, 0.84–1.01; *p* = 0.083) were not significantly associated with time to discharge. Cox proportional hazards modeling demonstrated that pediatric DKA patients with ESS had a significantly lower hazard of discharge compared with patients with normal thyroid function (HR = 0.31; 95% CI, 0.21–0.45; *p* < 0.001), indicating longer hospital stays.

We examined whether the effect of ESS on hospital stay varied by DKA severity by testing interaction terms between ESS and DKA severity categories. Neither the ESS × moderate DKA interaction (HR = 0.79; 95% CI, 0.38–1.63; *p* = 0.522) nor the ESS × severe DKA interaction (HR = 0.57; 95% CI, 0.19–1.73; *p* = 0.322) reached statistical significance. A likelihood ratio test comparing models with and without interaction terms confirmed that inclusion of interactions did not significantly improve model fit (χ^2^ = 1.06, df = 2, *p* = 0.590), indicating that the association between ESS and prolonged hospitalization is consistent across levels of DKA severity (Supplementary Table S1).

PICU admission was similarly associated with a reduced discharge hazard (HR = 0.49; 95% CI, 0.29–0.83; *p* = 0.008). With respect to DKA severity, both moderate DKA (HR = 0.51; 95% CI, 0.30–0.87; *p* = 0.014) and severe DKA (HR = 0.28; 95% CI, 0.14–0.57; *p* < 0.001) were associated with prolonged hospital stay compared with mild DKA. In contrast, age (HR = 0.98; 95% CI, 0.94–1.02; *p* = 0.267) and HbA1c (HR = 0.99; 95% CI, 0.89–1.09; *p* = 0.775) were not significantly associated with time to discharge (Table 3).

The global test (Schoenfeld residuals) indicated no significant violation across all covariates combined (*p* = 0.309). However, DKA severity showed evidence of non-proportionality (*p* = 0.035), indicating that its effect on discharge hazard varied over the follow-up period. In contrast, thyroid status (*p* = 0.715), age (*p* = 0.549), PICU admission (*p* = 0.137), and HbA1c (*p* = 0.859) all satisfied the proportional hazards assumption (Supplementary Figure S1).

The stratified model yielded results consistent with the primary analysis: ESS and PICU admission remained strong independent predictors of prolonged hospital stay (HR = 0.32; 95% CI, 0.22–0.47; *p* < 0.001) and (HR = 0.50; 95% CI, 0.30–0.84; *p* = 0.009), respectively. Age (HR = 0.98; 95% CI, 0.94–1.02; *p* = 0.269) and HbA1c (HR = 0.98; 95% CI, 0.88–1.09; *p* = 0.740) were not significant (Supplementary Table S2).

## 4. Discussion

ESS is an adaptive response to critical illness, characterized by alterations in thyroid hormone levels in the absence of intrinsic thyroid disease [25,26]. This study provides new insights into the clinical significance of ESS in pediatric patients presenting with DKA, highlighting its association with prolonged hospital stay.

### 4.1. Summary of Main Findings

In this study of 176 pediatric patients with DKA with no history of thyroid disease, 63.6% fulfilled the criteria of ESS. Children with ESS were younger, had lower weight and BMI, and presented with slightly more severe metabolic acidosis compared to those in the non-ESS groups. The hormonal profile of patients with ESS showed significantly lower free T3 and free T4, although TSH remained within the normal range. All cases with impaired consciousness were only in the ESS group. Participants with ESS experienced significantly prolonged hospital stays, with over a quarter staying five days or more. Multivariable Cox regression analyses demonstrated that ESS, PICU admission, and DKA severity (moderate and severe) were independent predictors of prolonged hospital stay, while age and glycemic control were not significant predictors. These findings highlight the clinical relevance of ESS as a potential marker of disease severity and prolonged hospitalization in pediatric DKA.

In this study, children who developed ESS were significantly younger than those who did not. However, age was not significantly associated with hospital stay duration in the Kaplan–Meier analysis or Cox regression. In contrast, other studies have reported the opposite pattern. For example, among 396 adults with diabetic ketosis/ketoacidosis, the ESS group had a higher mean age than the non-ESS group (60.5 ± 18.1 vs. 53.3 ± 17.7 years; *p* < 0.001) [19]. Similarly, in patients with COVID-19, those with ESS were older than patients without ESS (median age 58 vs. 41 years; *p* < 0.001) [27].

The finding that younger children were more prone to developing ESS may be due to an increased physiological sensitivity to metabolic stress. First, younger children have higher baseline metabolic rates and greater energy requirements per unit of body weight. This may exacerbate the catabolic state during DKA [28,29]. Second, the immature hypothalamic–pituitary–thyroid axis can be more easily disrupted by inflammatory cytokines, possibly leading to inadequate thyroid hormone responses [25]. Third, the limited glycogen storage reduces their metabolic adaptation capability compared with older children and adolescents, further increasing vulnerability under stress [30]. In contrast, in adult populations, older age is frequently associated with ESS, likely because aging is accompanied by multimorbidity, frailty, and reduced physiological reserves—factors that heighten susceptibility to non-thyroidal illness patterns [31]. Therefore, age appears to influence ESS risk in opposite directions depending on whether the affected population is pediatric or adult, highlighting the importance of considering age-specific physiology when interpreting thyroid hormone abnormalities in acute illness.

In the current study, there was a statistically significant difference between the ESS and non-ESS groups in terms of disturbed consciousness, with a higher prevalence observed in the ESS group. However, there is a scarcity of literature assessing neurological complications associated with ESS. A study by Yu et al. [32] found that ESS was a significant predictor of in-hospital stroke-associated pneumonia (iSAP) during the acute phase of ischemic stroke. Patients with ischemic stroke and ESS exhibited significantly higher white blood cell counts upon admission compared to those with normal T3 levels, further supporting the predictive value of ESS for iSAP. T3 plays a pivotal role in immune function, influencing the activity of various immune cells [33]. These findings suggest that ESS may not only reflect systemic illness severity but could also be associated with neurological and immune complications. However, the exact mechanisms need to be further studied. Consequently, further research is required to reveal whether thyroid hormone changes directly contribute to organ dysfunction in pediatric DKA or merely serve as a marker of illness severity.

This study found a significant association between ESS and prolonged hospital stays in children with DKA. Kaplan–Meier survival analysis revealed that pediatric patients with ESS experienced significantly longer durations of hospitalization compared with those who had normal thyroid function. This finding was confirmed in Cox proportional hazards regression analysis, in which ESS emerged as a strong, independent predictor of prolonged hospitalization. This independent effect suggests that ESS is not merely an epiphenomenon of severe illness; it may be a marker of a slower clinical recovery, reflecting a greater physiological insult or a diminished capacity to mount a rapid recovery. A similar association between ESS and clinical severity has been documented in other acute conditions; for example, low serum T3 levels have been strongly associated with prolonged hospital stays, increased need for PICU admission, and higher rates of mechanical ventilation among patients with acute heart failure [9,26]. Therefore, the diagnosis of ESS at admission could be a valuable tool for clinicians. It would allow more accurate risk stratification, informed discussions with families, and proactive discharge planning.

Metabolic acidosis is one of the most commonly diagnosed acid–base disorders among children admitted to the PICU. In the current study, duration of hospital stay was negatively associated with pH, indicating that children with more severe acidosis experienced longer recovery times. Multivariable Cox regression analysis demonstrated that the severity of DKA—a direct consequence of metabolic acidosis—was an independent predictor of prolonged hospitalization. Specifically, compared to children with mild DKA, those with moderate DKA had a 49% lower discharge hazard (HR = 0.51; *p* = 0.014), and those with severe DKA had a 72% lower hazard of discharge (HR = 0.28; *p* < 0.001). However, assessment of the proportional hazards assumption revealed that the effect of DKA severity on discharge hazard varied over time. Sensitivity analysis using a stratified Cox model with DKA severity as a stratification factor confirmed the robustness of our primary findings: ESS and PICU admission remained strong independent predictors of prolonged hospital stay. The consistency of effect estimates between the standard and stratified models underscores the validity of our conclusions despite the time-varying nature of DKA severity. Likewise, studies reported a significant association between acidosis severity and mortality [34]. On the other hand, a study conducted by Gutgold et al. [35] did not find an association between acidosis severity and survival. While Ravanagomagan et al. [36] found that acidosis was significantly associated with mortality but not with prolonged hospital stay. These differences highlight the complexity of acidosis as a prognostic indicator and suggest that its impact on outcomes like mortality and length of stay may differ across different patient populations, clinical backgrounds, or healthcare settings.

### 4.2. Implications of the Study

The findings of this study have important clinical and research implications. First, identifying patients with ESS at admission could alert clinicians to a higher risk of neurological complications and a prolonged clinical course, prompting more intensive monitoring and allowing for a more realistic setting of expectation with families. Routine measurement of thyroid function in children presenting with moderate to severe DKA could serve as a risk-stratification tool, though this remains hypothesis-generating at present. Validation of the current study findings in independent prospective cohorts before any clinical implementation is recommended. Second, the strong, independent association between ESS and length of stay could inform resource allocation and discharge planning.

### 4.3. Strengths and Limitations

Based on our knowledge, this study is the first to study the effect of ESS on the duration of hospital stay among children with DKA. The use of age-standardized definitions for DKA and ESS enhances the robustness and credibility of the results. Furthermore, survival analysis provides strong evidence regarding the length of hospital stay. Nonetheless, the retrospective design restricts the ability to infer causality, and some data may be affected by documentation bias or missing values. Furthermore, reverse T3 (rT3) level—a classical marker of ESS—was not included as a part of routine clinical care at our institution. However, rT3 measurement is rarely performed in clinical practice and is not required for ESS diagnosis when the characteristic FT3/TSH pattern is met [33]. Additionally, although thyroid function tests were obtained at presentation, the exact timing from DKA onset to blood sampling could not be precisely determined due to the retrospective design, which may introduce variability in the measured hormone levels. Lastly, because the research was conducted in a single geographic area using single measures, the generalization of the findings to other populations may be limited.

## 5. Conclusions

ESS is frequently diagnosed in pediatric patients with DKA and is associated with prolonged hospital stay and impaired consciousness. ESS serves as an independent predictor of delayed discharge, highlighting its potential utility as a clinical marker for identifying children at increased risk of unfavorable short-term outcomes during DKA episodes. Routine assessment of thyroid function at the time of hospital admission may facilitate more individualized monitoring and informed clinical decision-making, particularly in patients who present with severe metabolic derangement. However, given the retrospective design, these findings should be considered hypothesis-generating. Prospective multicenter studies are needed to elucidate the underlying pathophysiological mechanisms of ESS in pediatric DKA, investigate whether serial thyroid function testing offers dynamic prognostic information, and determine if supportive interventions could modulate the course of ESS and enhance recovery.

## Figures and Tables

**Table 1:** Clinical and biochemical characteristics by ESS classification.

Variable	Level	Overall (*n* = 176)	ESS (*n* = 112, 63.6%)	Normal (*n* = 64, 36.4%)	*p*-Value
Age (years)	Median [IQR]	12.0 [9.0, 15.0]	10.0 [6.5, 13.5]	14.0 [11.5, 16.0]	<0.001
Sex	Female	80 (45.5%)	48 (42.9%)	32 (50.0%)	0.4
	Male	96 (54.5%)	64 (57.1%)	32 (50.0%)	
Weight (kg)	Median [IQR]	36.5 [26.0, 45.0]	31.0 [20.5, 44.5]	40.5 [34.5, 49.5]	<0.001
BMI (kg/m^2^)	Median [IQR]	19.0 [17.0, 20.0]	18.0 [16.5, 20.0]	19.0 [17.5, 20.5]	0.007
pH	Mean ± SD	7.2 ± 0.1	7.1 ± 0.1	7.2 ± 0.1	0.016
HCO_3_ (mmol/L)	Mean ± SD	10.7 ± 2	10.5 ± 2.1	11.1 ± 1.9	0.06
Blood glucose at diagnosis (mg/dL)	Mean ± SD	486.9 (67.5)	482.7 (68.5)	494.2 (65.6)	0.2
HbA1c (%)	Mean ± SD	11.4 (1.6)	11.5 (1.5)	11.2 (1.7)	0.15
TSH (mIU/L)	Median [IQR]	2.1 [1.1, 3.1]	1.8 [1.1, 2.9]	2.7 [1.7, 3.2]	<0.001
Free T4 (pmol/L)	Median [IQR]	14.0 [11.5, 14.9]	12.0 [10.7, 14.1]	14.4 [14.0, 16.2]	<0.001
Free T3 (pmol/L)	Median [IQR]	3.7 [2.3, 4.8]	2.4 [2.0, 3.4]	5.1 [4.2, 5.5]	<0.001

Data were presented as mean ± SD for parametric variables, median [IQR] for non-parametric variables, or *n* (%) for categorical variables. *p*-values were calculated using the independent samples *t*-test for parametric variables (pH, HCO_3_, blood glucose, HbA1c), the Mann–Whitney U test for non-parametric variables (age, weight, BMI, TSH, free T4, free T3), and the Chi-squared test for categorical variables (Sex). *p*-values < 0.05 were considered statistically significant.

**Table 2:** Clinical outcomes by ESS status (*n* = 176).

Characteristic	Level	Overall *n* = 176	Non-ESS *n* = 64	ESS *n* = 112	*p*-Value
Hospital stays (days)	2	6 (3.4%)	4 (6.3%)	2 (1.8%)	<0.001
	3	64 (36.4%)	34 (53.1%)	30 (26.8%)	
	4	76 (43.2%)	26 (40.6%)	50 (44.6%)	
	5	22 (12.5%)	0 (0.0%)	22 (19.6%)	
	6	6 (3.4%)	0 (0.0%)	6 (5.4%)	
	7	2 (1.1%)	0 (0.0%)	2 (1.8%)	
	Mean ± SD	3.8 ± 0.9	3.3 ± 0.6	4.1 ± 1.0	<0.001
Level of consciousness	Normal	166 (94.3%)	64 (100.0%)	102 (91.1%)	0.034
	Disturbed	10 (5.7%)	0 (0.0%)	10 (8.9%)	
Dehydration grade	Mild	71 (40.3%)	30 (46.9%)	41 (36.6%)	0.2
	Moderate	79 (44.9%)	28 (43.8%)	51 (45.5%)	
	Severe	26 (14.8%)	6 (9.4%)	20 (17.9%)	
Electrolyte abnormalities	No	92 (52.3%)	30 (46.9%)	62 (55.4%)	0.4
	Yes	84 (47.7%)	34 (53.1%)	50 (44.6%)	
Infection present	No	76 (43.2%)	30 (46.9%)	46 (41.1%)	0.6
	Yes	100 (56.8%)	34 (53.1%)	66 (58.9%)	
PICU admission required	No	84 (47.7%)	28 (43.8%)	56 (50.0%)	0.5
	Yes	92 (52.3%)	36 (56.3%)	56 (50.0%)	
Mortality	No	176 (100.0%)	64 (100.0%)	112 (100.0%)	–

Data were presented as *n* (%) for categorical variables and mean ± standard deviation for the continuous variable ‘hospital stay’. For all other categorical variables, *p*-values were calculated using Pearson’s Chi-squared test. The *p*-value for ‘mortality’ was not applicable (–) as there were no events in either group.

**Table 3:** Hazard ratios for time to discharge from the hospital: Cox proportional hazards model.

Variable	Univariate HR (95% CI)	*p*-Value	Multivariate HR (95% CI)	VIF	*p*-Value
ESS (vs. non-ESS)	0.39 (0.27–0.54)	<0.001	0.31 (0.21–0.45)	1.31	<0.001
Age (per year)	1.03 (0.99–1.07)	0.096	0.98 (0.94–1.02)	1.25	0.267
PICU admission	0.34 (0.25–0.48)	<0.001	0.49 (0.29–0.83)	2.36	0.008
HbA1c (per 1%)	0.92 (0.84–1.01)	0.083	0.99 (0.89–1.09)	1.18	0.775
Moderate DKA (vs. mild)	0.33 (0.23–0.46)	<0.001	0.51 (0.30–0.87)	2.45	0.014
Severe DKA (vs. mild)	0.16 (0.10–0.26)	<0.001	0.28 (0.14–0.57)	2.28	<0.001

HR = hazard ratio; CI = confidence interval; ESS = Euthyroid Sick Syndrome; PICU = Pediatric Intensive Care Unit; HbA1c = glycated hemoglobin. HR < 1 indicates a slower rate of discharge, corresponding to a longer hospital stay. VIF values were calculated to assess multicollinearity among predictors; values < 5 indicate no significant multicollinearity. Model statistics: likelihood ratio, Wald, and log-rank tests all *p* < 0.001; concordance index = 0.853 (excellent predictive accuracy); explained variation R^2^ = 0.441; Akaike information criterion (AIC) = 1384.73.

## Data Availability

Data is uploaded as a Supplementary File attached to this manuscript.

## Supplementary Materials

The following supporting information can be downloaded at: https://www.mdpi.com/article/10.3390/jcm15072501/s1. Figure S1: Time-varying effects of clinical covariates on hospital outcomes: a Schoenfeld residual analysis; Table S1: Predictors of time to hospital discharge (stratified by DKA severity); Table S2: Predictors of time to hospital discharge (stratified by DKA severity).

## Abbreviations

The following abbreviations are used in this manuscript:

BMI	Body Mass Index
CDC	Centers for Disease Control and Prevention
CI	Confidence Interval
CKD	Chronic Kidney Disease
COPD	Chronic Obstructive Pulmonary Disease
DKA	Diabetic Ketoacidosis
ESS	Euthyroid Sick Syndrome
FT3	Free Triiodothyronine
FT4	Free Thyroxine
HbA1c	Glycated Hemoglobin
HLA	Human Leukocyte Antigen
HR	Hazard Ratio
PICU	Pediatric Intensive Care Unit
IDF	International Diabetes Federation
iSAP	In-Hospital Stroke-Associated Pneumonia
IQR	Interquartile Range
ISPAD	International Society for Pediatric and Adolescent Diabetes
NTI	Non-Thyroidal Illness
rT3	Reverse Triiodothyronine
SD	Standard Deviation
T1DM	Type 1 Diabetes Mellitus
T3	Triiodothyronine
T4	Thyroxine
TSH	Thyroid-Stimulating Hormone
WHO	World Health Organization
WBC	White Blood Cell (count)
