# Supplementary material for: Euthyroid Sick Syndrome as a Predictor of Hospital Stay in Pediatric Diabetic Ketoacidosis
Source: J Clin Med. 2026 Mar 25;15(7):2501. doi: 10.3390/jcm15072501 (PMC13073428; doi:10.3390/jcm15072501)
Supplement: Supplementary file 1 [file jcm-15-02501-s001.zip › jcm-4205783-supplementary.pdf]

## Supplementary files

This analysis utilized Schoenfeld residual plots to evaluate the proportional hazards assumption for key covariates — thyroid status, age, ICU admission, glycemic control, and DKA severity — over the follow-up period (approximately 3.1 to 5.8 days). The global test indicated no significant violation of the proportional hazards assumption across all covariates combined ($p = 0.3092$). However, individual tests identified DKA severity as exhibiting a statistically significant time-varying effect ($p = 0.0346$), suggesting that its impact on the outcome (e.g., risk of discharge) changes over time. In contrast, thyroid status ($p = 0.7152$), age ($p = 0.5492$), ICU admission ($p = 0.1365$), and glycemic control ($p = 0.859$) showed no evidence of non-proportionality. The Beta(t) curves support these findings: most covariates remain near zero with stable confidence bands, whereas DKA severity displays a pronounced dip around day 4 before rising again, indicating a non-constant effect. This pattern may reflect greater influence of DKA severity early in hospitalization, with a transient reduction in effect mid-course, followed by renewed impact later in patients with slower recovery. These results imply that standard Cox models assuming constant hazard ratios for DKA severity may be mis specified, and incorporating time-dependent effects for this variable could enhance both predictive accuracy and clinical interpretability.

Time to discharge hospital was analyzed using Cox proportional hazards regression. The proportional hazards (PH) assumption for each covariate was evaluated using Schoenfeld residuals and corresponding global and variable-specific tests. Scaled Schoenfeld residual plots were visually inspected to assess time-dependent changes in regression coefficients ($\beta(t)$) over the follow-up period. Because DKA severity demonstrated evidence of non-proportional hazards ($p = 0.0346$), while the global test was not significant ($p = 0.3092$), a stratified Cox regression model was fitted with DKA severity entered as a stratification variable. This approach allows the baseline hazard to vary across DKA severity categories without estimating a single constant hazard ratio for this variable, thereby accounting for its time-varying effect while preserving valid estimates for the remaining covariates.

The final multivariable model therefore included:

- thyroid status (ESS vs normal)
- age (per year)
- ICU admission
- HbA1c (per 1%)

and was stratified by DKA severity.

Hazard ratios (HRs) with 95% confidence intervals (CIs) were reported. Model performance was assessed using the likelihood ratio test, Wald test, log-rank test, concordance index (C-statistic), and Akaike information criterion (AIC). In the time-to-event analysis, the proportional hazards assumption was assessed using Schoenfeld residuals. The global test showed no significant violation; however, DKA severity exhibited a significant time-varying effect, indicating that its impact on discharge hazard was not constant over time. Therefore, DKA severity was included as a stratification factor in the final Cox model.

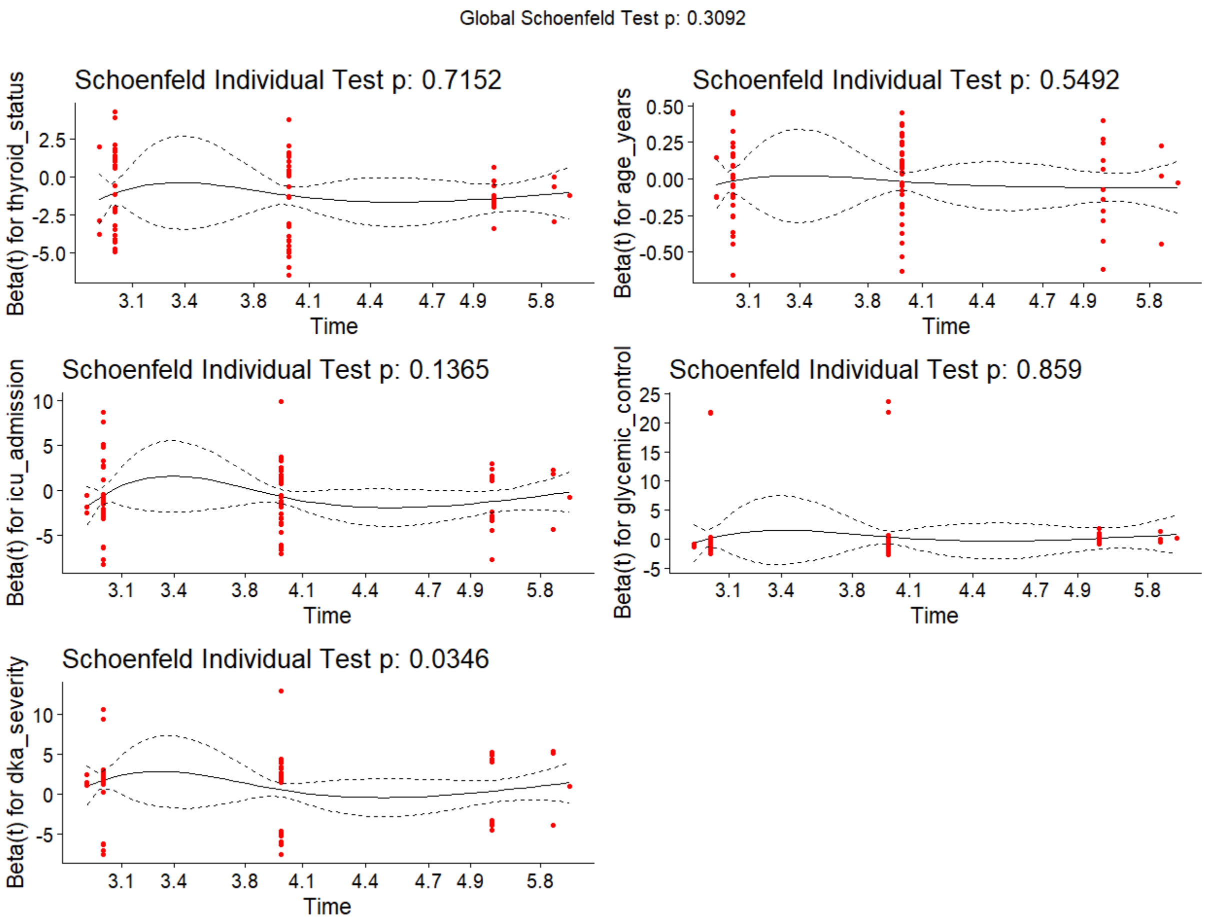


**Supplementary figure S1: Time-varying effects of clinical covariates on hospital outcomes: a Schoenfeld residual analysis**

**Supplementary table S1. Evaluation of interaction between Euthyroid Sick Syndrome and DKA severity on time to hospital discharge**

| Interaction Term | Hazard Ratio (HR) | 95% Confidence Interval | p-value |
|---|---|---|---|
| **ESS × Moderate DKA (vs Mild)** | 0.79 | 0.38 – 1.63 | 0.522 |
| **ESS × Severe DKA (vs Mild)** | 0.57 | 0.19 – 1.73 | 0.322 |

*Abbreviations: ESS, Euthyroid Sick Syndrome; DKA, diabetic ketoacidosis; HR, hazard ratio; CI, confidence interval; df, degrees of freedom*

**Model fit comparison:** Likelihood ratio test (interaction model vs. main effects model): $\chi^2 = 1.06$, df = 2, p = 0.590

After adjustment for DKA severity, ESS and ICU admission remained independent predictors of delayed hospital discharge. Patients with ESS had a 68% lower discharge hazard compared with euthyroid patients (HR = 0.32; 95% CI 0.22–0.47; p < 0.001). ICU admission was also associated with a significantly reduced discharge rate (HR = 0.50; 95% CI 0.30–0.84; p = 0.009). Age and HbA1c were not significantly associated with time to discharge. The model demonstrated good overall performance (likelihood ratio p < 0.001) with acceptable discrimination (C-index = 0.72).

**Supplementary Table S2: Predictors of time to hospital discharge (stratified by DKA severity)**

| Variable | HR | 95% CI | p-value |
|---|---|---|---|
| ESS (vs Normal) | **0.32** | 0.22 – 0.47 | <0.001 |
| Age (per year) | 0.98 | 0.94 – 1.02 | 0.269 |
| ICU Admission | **0.50** | 0.30 – 0.84 | 0.009 |
| HbA1c (per 1%) | 0.98 | 0.88 – 1.09 | 0.740 |

**Model fit statistics**

| Metric | Value |
|---|---|
| Likelihood ratio test | 35.19 (p < 0.001) |
| Wald test | 36.01 (p < 0.001) |
| Log-rank test | 38.02 (p < 0.001) |
| Concordance (C-index) | 0.72 |
| AIC | 1103.32 |
